# Supplementary material for: Splice-Junction-Based Mapping of Alternative Isoforms in the Human Proteome
Source: Cell Rep. Author manuscript; Available in PMC 2020 Jan 15. (PMC6961840; doi:10.1016/j.celrep.2019.11.026)
Supplement: 2 [file NIHMS1546469-supplement-2.zip › DF1/PXD010154/Testis/Testis_13_MAP4_MYHDDDLADLVFPSSATADTSIFAGQNDPLKDSYVPLELAK.pdf]

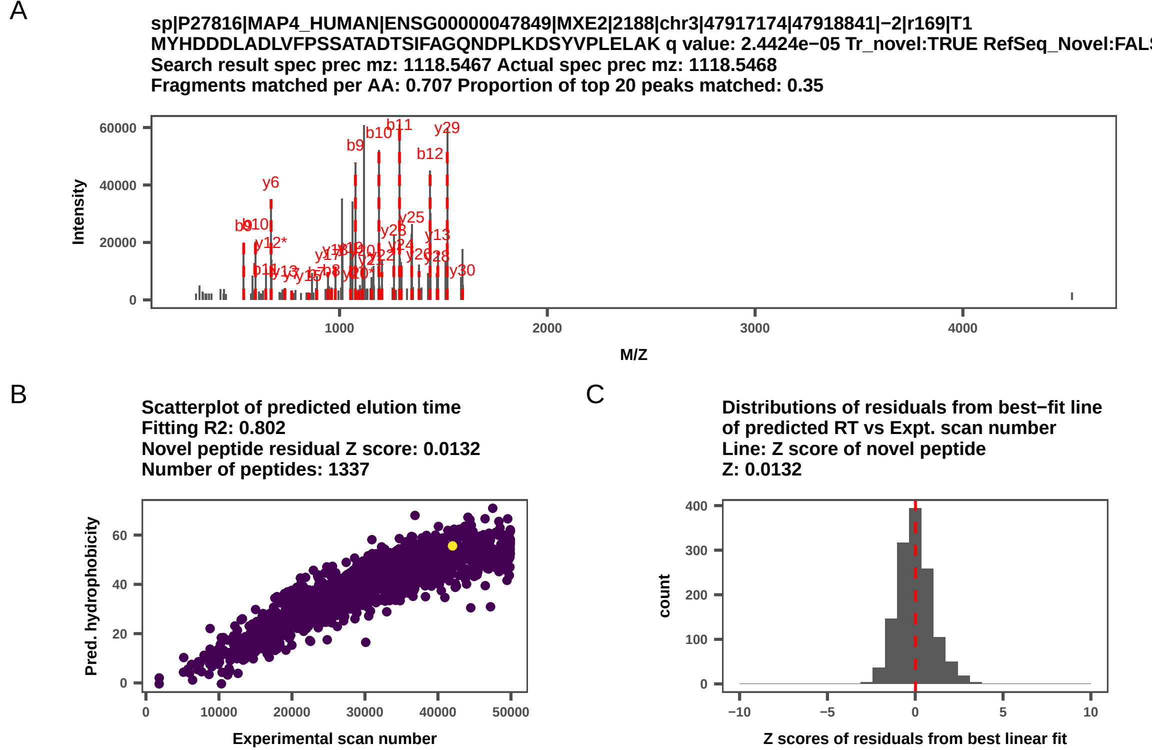
A
sp|P27816|MAP4_HUMAN|ENSG00000047849|MXE2|2188|chr3|47917174|47918841|−2|r169|T1
MYHDDDLADLVFPSSATADTSIFAGQNDPLKDSYVPLELAK q value: 2.4424e−05 Tr_novel:TRUE RefSeq_Novel:FAL
Search result spec prec mz: 1118.5467 Actual spec prec mz: 1118.5468
Fragments matched per AA: 0.707 Proportion of top 20 peaks matched: 0.35
Intensity
60000
40000
20000
0
b9
b10
b11
y29
b12
y6
y25
y23
y13
y12*
y24
y22
y21
y26
y28
y30
y17
1000
2000
3000
4000
M/Z
B
Scatterplot of predicted elution time
Fitting R2: 0.802
Novel peptide residual Z score: 0.0132
Number of peptides: 1337
Pred. hydrophobicity
60
40
20
0
0
10000
20000
30000
40000
50000
Experimental scan number
C
Distributions of residuals from best−fit line
of predicted RT vs Expt. scan number
Line: Z score of novel peptide
Z: 0.0132
count
400
300
200
100
0
−10
−5
0
5
10
Z scores of residuals from best linear fit
